# Supplementary material for: Optimized molecule detection in localization microscopy with selected false positive probability
Source: Nat Commun. 2025 Jan 11;16:601. doi: 10.1038/s41467-025-55952-5 (PMC11724879; doi:10.1038/s41467-025-55952-5)
Supplement: Supplementary file 1 — Supplementary Information [file 41467_2025_55952_MOESM1_ESM.pdf]

Supplementary Information

Optimized Molecule Detection in Localization Microscopy with Selected False Positive Probability

Miroslav Hekrdla[1*], David Roesel[1], Niklas Hansen[1,2], Soumya Frederick[1,2], Khalilullah Umar[1,3], Vladimíra Petráková[1*]

[1]J. Heyrovský Institute of Physical Chemistry, Czech Academy of Sciences, Dolejškova 3, 18223, Prague, Czech Republic
[2]Department of Physical Chemistry, University of Chemistry and Technology, Technická 5, 16628, Prague, Czech Republic
[3]Faculty of Biomedical Engineering, Czech Technical University in Prague, Nám. Sítná 3105, 27201, Kladno, Czech Republic
[*]Correspondence to: miroslav.hekrdla@jh-inst.cas.cz, vladimira.petrakova@jh-inst.cas.cz

**Supplementary Note 1**	Brief Overview of Molecule Detection Methods in SMLM
**Supplementary Note 2**	Software Implementation
**Supplementary Note 3**	Relation Between Poisson Matched Filter (PMF) and Matched Filter (MF)
**Supplementary Note 4**	Molecule Detection in Pixel-Independent Poisson-Gaussian Noise: CCD Camera
**Supplementary Note 5**	Molecule Detection in Pixel-Dependent Poisson-Gaussian Noise: sCMOS Camera
**Supplementary Note 6**	Probabilistic Thresholding for Stochastic Noise Model of EMCCD Camera
**Supplementary Note 7**	Relationships Between Molecule Detection and Radar Systems
**Supplementary Note 8**	Detection Performance in 3D Using Simulated Multiplane
**Supplementary Note 9**	Experimental Dataset
**Supplementary Note 10**	Computational Complexity
**Supplementary Note 11**	Comparison to Deep Learning Methods
**Supplementary Note 12**	Extension to Multi-Molecule Detection Problem
**Supplementary Note 13**	Filter Kernel Visualization
**Supplementary Note 14**	Generalization for Molecule Detection in 3D
**Supplementary Figure 9**	Performance of Detection Methods on 2D MT3 Dataset
**Supplementary Figure 10**	Example Signal with a Uniform Background
**Supplementary Figure 11**	Jaccard Index Evaluation of Simulations with Uniform Background and Poisson Noise
**Supplementary Figure 12**	Performance over Simulated Microtubule Dataset
**Supplementary Figure 13**	Immobilization of DNA nanostructures for SMLM measurements
**Supplementary Figure 14**	Gold Nanoparticles Attached to the DNA Origami
**Supplementary Figure 15**	Impact of Integer Position Approximation
**Supplementary Figure 16**	Example Signal with a Varying Background
**Supplementary Table 5**	List of Synonyms

Supplementary Note 1: Brief Overview of Molecule Detection Methods in SMLM

Here we discuss the great variety of the methods used for molecule detection in Single-Molecule Localization Microscopy (SMLM). The molecule detection in SMLM [1], [2], [3] is closely related to the problem of Single-Particle Tracking (SPT) [4], and shares many concepts from fluorescence object detection in microscopy image analysis [5], [6] and image segmentation in cell analysis [7]. A complete overview of all detection methods in SMLM and related fields is beyond the scope of this work. In this section, we present the most commonly encountered concepts. The general framework for molecule detection is shown in supplementary Figure 1. The first processing operation is raw image filtering, which aims to suppress noise, enhance the signal, and remove a slowly varying background or a combination of the above. The subsequent thresholding operation determines which pixels correspond to the detected molecules and which to the background. The output as well as the filtering and thresholding operations are collectively referred to as image segmentation. The segmentation is further processed by an algorithm that searches for a local maximum. We use the term detection for the sequence of filtering, thresholding, and local maximum search operations. The localization algorithm in the neighborhood of each detection then determines the exact sub-pixel location, which we denote as localization. In this work we focus on the detection.

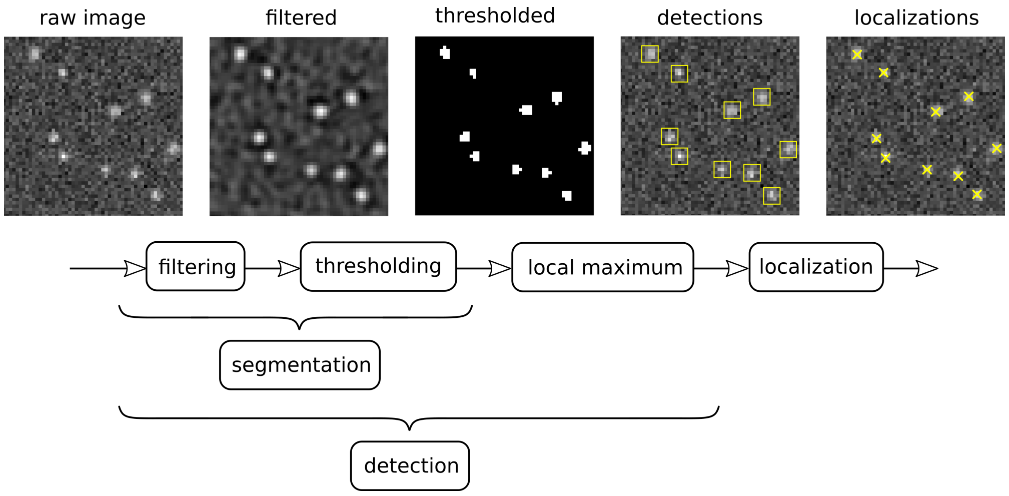

Supplementary Figure 1: General framework for the methods of SMLM molecule detection.

We divide filtration methods into linear, non-linear, and those based on mathematical morphology. The linear methods include the mean filter (box filter), Gaussian filter, lowpass and bandpass filters [1], [6], filter compositions such as Laplacian of Gaussian (LoG), Difference of Gaussians (DoG), Difference of Arithmetic means (DoA), Lowered Gaussian (LG) and similar, and filters that are based on wavelet transform (e.g. à trous B-Spline (BS), discrete wavelet transform). Mean and Gaussian averaging filters are used for noise suppression or background estimation. If these operations are combined, we obtain a DoG-type filter or its computationally faster equivalent DoA filter. A lowpass filter can be used for noise suppression and a bandpass filter can be used to filter out a slowly changing background. BS filters that simultaneously suppress noise, enhance the signal, and remove the background can also be thought of as bandpass filtering [8]. The spot-enhancing LoG filter is proposed in [9] based on the additive colored Gaussian noise approximation of measured noise for which the optimal filter is close to the LoG filter. List of frequently occurring filters, their references, and the SMLM software availability is summarized in Table 1 in the manuscript. Non-linear filters include median filters and other more advanced image processing methods for noise suppression such as bilateral filtering, non-local means, Block-Matching and 3-Dimensional (BM3D), and many others [10]. Morphological operations such as the top-hat filter and the h-dome transform appear in the context of molecule detection [5].

In the SMLM and SPT challenges [1], [2], [4], there are various approaches to determine the detection threshold, often based on methods from classical image processing such as the Otsu's threshold, 3 sigma noise level threshold, or a threshold given by certain Signal-to-Noise Ratio (SNR).

In addition to the techniques mentioned so far, closely related are methods of image segmentation

based on traditional image processing methods such as watershed transform, edge-based segmentation, region-based segmentation, active contours, and many others [11], [12]. At the same time, we cannot ignore the ever-improving machine learning-based segmentation methods [5], [6] and others, especially the dominating deep learning methods that have seen tremendous advances in the last decade [13]. The detection task in SMLM can also be viewed through a computer vision perspective as an object detection problem with a long history and a variety of existing methods, including deep learning methods [14].

Supplementary Note 2: Software Implementation

All performance evaluation simulations are written in the Python programming language and their source code is publicly available as a set of Jupyter notebooks on our GitLab repository at https://gitlab.com/-VladkaPetrakova/petrakova_group/-/tree/main/mirek/publication/molecule_detection. Partial analyses of various filters of B-spline wavelet, Laplacian of Gaussian, difference of Gaussians, and difference of arithmetic means written in Mathematica are also available there.

In programming the receiver operating characteristic simulations, we took advantage of the standard SciPy library [15], especially for the inversion of complementary cumulative distribution function (`scipy.stats.norm.isf`, `scipy.stats.poisson.isf`), robust estimation by inter quantile range (`scipy.stats.iqr`), local filtering with an arbitrary kernel shape (`scipy.ndimage.convolve` , `scipy.signal.convolve`, `scipy.ndimage.median_filter`, `scipy.ndimage.generic_filter`), and for the assignment problem (`scipy.optimize.linear_sum_assignment`).

Supplementary Note 3: Relation Between Poisson Matched Filter (PMF) and Matched Filter (MF)

Interestingly, for a low ratio of $^a/_b$, we can linearly approximate natural logarithm in PMF kernel by $\ln(1+x) \simeq x,\ x \ll 1$ so that

$$w_{mn} = \ln\left(1 + \frac{a}{b} s_{mn}(\sigma)\right) \simeq \frac{a}{b}\, s_{mn}(\sigma). \quad (1)$$

This modifies the GLRT test to yield

$$T(\mathbf{R}) = \sum_{mn} r_{mn} w_{m-\hat{m}_0, n-\hat{n}_0}(\hat{a}, \hat{b}, \hat{\sigma}) \simeq \sum_{mn} r_{mn} \frac{\hat{a}}{\hat{b}} s_{m-\hat{m}_0, n-\hat{n}_0}(\hat{\sigma}) \overset{H_1}{>} \tau' \quad (2)$$

simplifying by new threshold τ'' to the test

$$T'(\mathbf{R}) = \sum_{mn} r_{mn} s_{m-\hat{m}_0, n-\hat{n}_0}(\hat{\sigma}) \overset{H_1}{>} \frac{\hat{b}}{\hat{a}} \tau' = \tau''. \quad (3)$$

The test (3) correlates the raw image with the PSF signal model. This type of detection is well-known Matched Filtering (MF) sometimes denoted as correlation filtering, pattern matching, or template matching. In the case of the Gaussian PSF model, the MF is equivalent to Gaussian filtering, or Gaussian smoothing. Note, that in the test (3), the unknown parameters a, b do not need to be estimated because they are part of a new detection threshold τ'' that is to be determined by P_{FP}.

The result is intuitive, because for a low $^a/_b$ ratio the signal model is closer to the Additive White Gaussian Noise (AWGN), where MF is known to be the optimal filter [16]. Supplementary Figure 2 compares MF to PMF for several values of signal-to-background ratio $^a/_b$. The lower the ratio $^a/_b$, the closer PMF is to MF. To implement the MF, the estimation of parameters can be done accordingly to PMF, but only σ needs to be estimated.

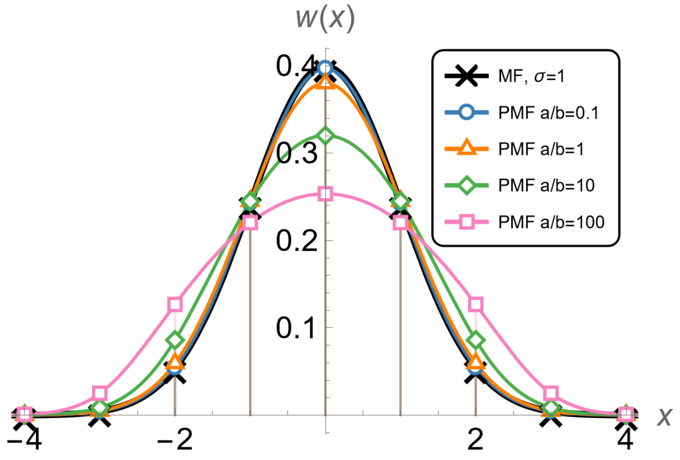

Supplementary Figure 2: Comparison of PMF with MF for several values of $^a/_b$ ratio, for visualization purposes in 1D. Markers correspond to the sampled filter values w_n. Continuoues plots show generalization to $\mathbb{R}$, where continuous PMF is $w(x) = {}^1/_C \ln(1 + {}^a/_b\, s(x))$ with C such that $\int_{-\infty}^{\infty} w(x)\mathrm{d}x = 1$. We see that PMF is close to MF for ratio $^a/_b = 0.1$ and lower.

Supplementary Note 4: Molecule Detection in Pixel-Independent Poisson-Gaussian Noise: CCD Camera

In this section, we focus on deriving the optimal detection filter in pixel-independent Poisson-Gaussian noise model related to the dominant noise sources in the CCD camera.

4.1 GLRT in Poisson-Gaussian Noise

Let us consider a detection problem of a PSF signal in Poisson-Gaussian noise which is a photon noise model that additionally takes into account, e.g., the read-out noise of the CCD camera. Such an image formation model corresponds to the following hypotheses

$$H_0 : \mathbf{R},\, [\mathbf{R}]_{mn} = r_{mn} \sim \mathscr{P}(b) + \mathscr{N}(0, \sigma_w^2), \tag{4}$$

$$H_1 : \mathbf{R},\, [\mathbf{R}]_{mn} = r_{mn} \sim \mathscr{P}(as_{m-m_0,n-n_0}(\sigma) + b) + \mathscr{N}(0, \sigma_w^2), \tag{5}$$

where the superposition of Poisson random variable and Gaussian random variable has PDF given by the convolution of the individual PDFs [17]. The resulting PDF is for reasonably high background (more than 5 photons and more as illustrated in Figure 15.12 in [17]) well approximated by Gaussian PDF as

$$H_0 : \mathbf{R},\, [\mathbf{R}]_{mn} = r_{mn} \sim \mathscr{N}(b, b + \sigma_w^2), \tag{6}$$

$$H_1 : \mathbf{R},\, [\mathbf{R}]_{mn} = r_{mn} \sim \mathscr{N}(x_{m-m_0,n-n_0}, x_{m-m_0,n-n_0} + \sigma_w^2), \tag{7}$$

where we have shorten the notation by symbol $x_{m-m_0,n-n_0} \triangleq as_{m-m_0,n-n_0} + b$. Unfortunately, the corresponding likelihood functions

$$p(\mathbf{R}|H_0) = \left[2\pi\left(b + \sigma_w^2\right)\right]^{-\frac{MN}{2}} \mathrm{e}^{-\frac{1}{2(b+\sigma_w^2)} \sum_{mn}(r_{mn}-b)^2}, \tag{8}$$

$$p(\mathbf{R}|H_1) = (2\pi)^{-\frac{MN}{2}} \left[\prod_{mn}\left(x_{m-m_0,n-n_0} + \sigma_w^2\right)\right]^{-\frac{1}{2}} \mathrm{e}^{-\frac{1}{2}\sum_{mn} \frac{\left(r_{mn}-x_{m-m_0,n-n_0}\right)^2}{x_{m-m_0,n-n_0}+\sigma_w^2}} \tag{9}$$

do not lead to a compact formula for the GLRT test.

4.2 GLRT Approximated by MF

GLRT is simplified, if we prioritize detection of the weak signal with a low signal-to-background ratio such that $as_{mn} \ll b + \sigma_w^2$. Under this condition, the GLRT is equivalent to MF filtering leading to the test

$$T(\mathbf{R}) = \sum_{mn} r_{mn} w_{m-\hat{m}_0,n-\hat{n}_0}(\hat{\sigma}) = \sum_{m,n=-L}^{L} r_{m+\hat{m}_0,n+\hat{n}_0} w_{mn} \overset{H_1}{>} \tau, \tag{10}$$

where filter weights w_{mn} equal to the sampled PSF signal $w_{mn} = s_{mn}$. Symbols $\hat{m}_0, \hat{n}_0, \hat{\sigma}_0$ are parameter estimates obtained as for PMF.

4.3 Probabilistic Thresholding in Poisson-Gaussian Noise

Similar to Poisson noise, we can derive probabilistic thresholding by analyzing a test statistic that is normally distributed for the null hypothesis with the following moments

$$E[T(\mathbf{R})|H_0] = E\left[\sum_{m,n=-L}^{L} r_{m+\hat{m}_0,n+\hat{n}_0} w_{mn} | H_0\right] = b \sum_{m,n=-L}^{L} w_{mn}, \tag{11}$$

$$\mathrm{var}[T(\mathbf{R})|H_0] = \mathrm{var}\left[\sum_{m,n=-L}^{L} r_{m+\hat{m}_0,n+\hat{n}_0} w_{mn} | H_0\right] = \sigma_b^2 \sum_{m,n=-L}^{L} w_{mn}^2, \tag{12}$$

where symbol $\sigma_b^2 \triangleq b + \sigma_w^2$ is variance of the Poisson background plus read-out AWGN noise. The probability of a false positive detection

$$P_{\mathrm{FP}} = Q\left(\frac{\tau - E[T(\mathbf{R})|H_0]}{\sqrt{\mathrm{var}[T(\mathbf{R})|H_0]}}\right) = Q\left(\frac{\tau - b\sum_{m,n=-L}^{L} w_{mn}}{\sigma_b \sqrt{\sum_{m,n=-L}^{L} w_{mn}^2}}\right) \tag{13}$$

and the corresponding probabilistic threshold

$$\tau = \hat{\sigma}_b \sqrt{\sum_{m,n=-L}^{L} w_{mn}^2}\, Q^{-1}(P_{\mathrm{FP}}) + \hat{b} \sum_{m,n=-L}^{L} w_{mn} \tag{14}$$

have a similar form to that of Poisson noise, except that now, additionally, the variance of the background $\hat{\sigma}_b^2$ must be estimated to determine the threshold.

For the thresholding calculation (14), we use an estimate of both moments (mean and variance), so we should correctly use the inverse of the CCDF function of the student's t-distribution. Since we always use at least 100 values for the estimation (the number of reference pixels of the adaptive thresholding kernel, see Figure 3d) in the manuscript), which is the number of degrees of freedom of the t-distribution, then using the inverse CCDF of the Gaussian distribution approximates the true CCDF well enough.

Supplementary Note 5: Molecule Detection in Pixel-Dependent Poisson-Gaussian Noise: sCMOS Camera

In this section, we focus on deriving the optimal detection filter in pixel-dependent Poisson-Gaussian noise model related to the dominant noise in the sCMOS camera.

For the SMLM method, an EMCCD camera is typically used because of its high sensitivity and negligible effective read-out noise. Nowadays, the alternative sCMOS camera technology is also gaining popularity due to its high sensitivity, small read-out noise and especially high read-out speed for large FOV. Although the read-out noise of the sCMOS camera is low, it is not negligible as in EMCCD, and instead of pure Poisson distributed noise, the noise is a Poisson-Gaussian mixture similar to a conventional CCD camera. Unlike a CCD camera, the noise in an sCMOS camera has different distribution parameters for each pixel, which is usually referred to as pixel-dependent read-out noise.

Since the read-out noise is independent of the signal being measured, its parameters can be measured and calibrated. For an sCMOS camera, we obtain an estimate of the offset and variance of the read-out noise of a single pixel by averaging dark images, and an estimate of the pixel gain by linear regression of images with different irradiance levels, see reference [18] for a more detailed description of the calibration method.

5.1 GLRT in Pixel-Dependant Poisson-Gaussian Noise

By subtracting the estimated offsets and dividing by the estimated gains at each pixel, we obtain an effective model of the signal generation similar to that of a CCD camera, see Supplementary Note 3, except that now each pixel has a different (though known) value of the noise variance σ_{mn}^2. Detection hypothesis correspond to (4) and (5) with σ_{mn}^2 as follows

$$H_0 : r_{mn} \sim \mathscr{P}(b) + \mathscr{N}(0, \sigma_{mn}^2), \tag{15}$$

$$H_1 : r_{mn} \sim \mathscr{P}(as_{m-m_0,n-n_0} + b) + \mathscr{N}(0, \sigma_{mn}^2), \tag{16}$$

A GLRT detector with these likelihood functions will not lead to a compact solution that is easy to implement. Similarly to the case of CCD camera, we obtain a simpler form that can be easily implemented as a linear filtering if we focus on the case where the background level is high enough that it can be approximated by a normal distribution ($b \gg 10$) and optimize the detector to detect a low signal-to-background ratio signal ($as_{m-m_0,n-n_0} \ll b + \sigma_{mn}^2$). The hypothesis simplifies to

$$H_0 : r_{mn} \sim \mathscr{N}(b, b + \sigma_{mn}^2), \tag{17}$$

$$H_1 : r_{mn} \sim \mathscr{N}(as_{m-m_0,n-n_0} + b, b + \sigma_{mn}^2). \tag{18}$$

5.2 Detection in Uncorrelated Additive Colored Gaussian Noise

The simplified detection problem (18) is in engineering literature ([16], Chapter 4) denoted as the detection in uncorrelated additive Gaussian noise with unequal variances or as the detection in uncorrelated Additive Colored Gaussian Noise (ACGN) as opposed to Additive White Gaussian Noise (AWGN). After similar derivation steps as in the section 3, we obtain the test statistic being equal to

$$T(\mathbf{R}) = \sum_{mn} \frac{r_{mn} s_{m-\hat{m}_0,n-\hat{n}_0}}{\sigma_{mn}^2}. \tag{19}$$

The test statistic is a Matched Filter (MF) applied on the inverse-variance weighted received signal r_{mn}/σ_{mn}^2. This operation is sometimes denoted as a noise pre-whitening generalized MF [16] or Whitened Matched Filter (WMF). The equations describing probabilistic thresholding and detection performance given in Section 3 apply, with the only difference that instead of the received signal, we now consider a pre-whitened received signal.

Supplementary Note 6: Probabilistic Thresholding for Stochastic Noise Model of EMCCD Camera

In the analysis and design of data processing algorithms for SMLM, simplified analytical and computationally less demanding statistical models of noise are often used, which consider only its dominant components. The literature refers to such models as practical data models [17]. For example, the Poisson photon (shot) noise is chosen as the baseline model [19], [20], under the assumption that other significant noise components such as dark noise and read-out noise are sufficiently suppressed in modern high-performance cameras. This is often applied for EMCCD camera, which allows to reduce the effective read-out noise to a small negligible value by the electron-multiplying gain register placed before the output amplifier.

However, this also implies that the whole amplification process is inherently statistically random. The distribution function of the constant signal especially for its low input values is heavily right-skewed violating the normally distributed noise assumption [3]. This means that under such conditions, the probabilistic thresholding (see Section 2.3 in the manuscript) which assumes a Poisson or normal distribution produces more false positive detections than the desired due to the strong right-tailedness of the true distribution. In order to strictly adhere to the desired false positive rate, the inverse CCDF function of the true EMCCD noise distribution needs to be used to calculate the probabilistic thresholding. Its analytical form is complex (see the derivation in reference [17], Chapter 15 and Appendix G), but in the practical case for high EM gain values it simplifies to an exponential distribution or a more general model using a gamma distribution as proposed in [21]. These models are practically useful because they are available in most standard statistical packages such as `scipy.stats` in Python.

Supplementary Figure 3 illustrates that the histogram of intensities from the data from SMLM software challenge 2016 [2] from the MT0_N2_LD dataset with EMCCD camera noise, high EM gain value, and low signal level is better described by the gamma distribution. In particular, it can be seen that the alternative Poisson and normal distributions do not achieve such a skewed right-tail, which then causes a higher than desired false positive detection rate. In this example, we use computationally simple non-iterative closed-form formulas for gamma parameter estimation proposed in [22].

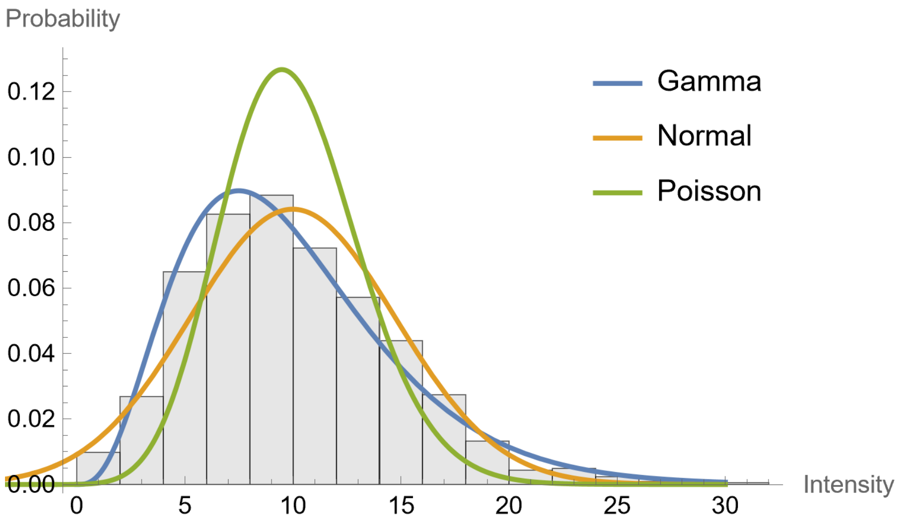

Supplementary Figure 3: Comparison of gamma, normal and Poisson distributions fitted to the background noise in MT0_N2_LD dataset taken from the SMLM software challenge 2016 [2]. Gamma distribution better captures the skewed right-tailedness of the true distribution which is mainly responsible for the rate of false positive detections.

Supplementary Note 7: Relationships Between Molecule Detection and Radar Systems

Detecting fluorescent molecules in SMLM microscopy has many parallels with the problem of target detection in radar, which was a major inspiration for this work. In the case of radar, the detection is usually performed in the two-dimensional space of the position and velocity of the target (Doppler shift), which is obscured by Rayleigh distributed noise [23]. The detection threshold is determined by the chosen false positive probability (denoted as false alarm rate), as we propose in this paper. The detection is simultaneously performed on a strongly non-uniform background, which is called radar clutter (analogous to a non-uniform fluorescence background in microscopy). Although the detection problem differs in part by the statistical model of signal generation, the signal detection theory (originally developed for radars) is general enough to be applicable in microscopy with Poisson or Poisson-Gaussian noise. The adaptive thresholding method is called CFAR detection in the radar context. Adaptive estimation using the local arithmetic mean with a doughnut-shaped filter (depicted in Figure 3d) in the manuscript) is called Cell Averaging (CA)-CFAR in the radar context. The term Ordered Statistic (OS)-CFAR is used to denote the robust local median filtering. In the context of radar detection, we also talk about sub-pixel refinement of position estimation (super-resolution in microscopy) and subsequent tracking similar to SMLM and SPT. More interconnections between microscopy and radar systems provides inspiring reference [24].

Supplementary Note 8: Detection Performance in 3D Using Simulated Multiplane

As a proof of concept for the application of detection theory in 3D SMLM, we built a simple Monte Carlo simulation for molecule detection in the multiplane method. Without loss of generality, we chose the simple Gaussian function as the 3D PSF model with a symmetric shape in the x-y axis and an elongated shape in the z-axis. The overall shape resembling a rugby ball is depicted in supplementary Figure 4. The specific definition used is

$$s_{lmn} = \frac{1}{2\pi\sigma_{xy}^2} \mathrm{e}^{-\frac{1}{2\sigma_{xy}^2}\left(m^2+n^2\right)} \frac{1}{\sqrt{2\pi\sigma_z^2}} \mathrm{e}^{-\frac{1}{2\sigma_z^2} l^2}, \tag{20}$$

where we chose the ratio between σ_{xy} and σ_z to follow theoretically ideal case of ratio between FWHM_{xy} and FWHM_z [25], which wields

$$\sigma_z = {}^{4}\!/\!_{\mathrm{NA}}\, \sigma_{xy} = 2.67\, \sigma_{xy}, \text{ for NA} = 1.5. \tag{21}$$

Detection ROC curves depicted in supplementary Figure 5 were measured based on a simulation where one randomly placed molecule with σ_{xy} equal to one pixel was always present in a 128x128x128 pixel 3D datacube. The average background level was 100 photons and the signal level was 612.5 photons. From the previously mentioned detection filters, we selected the most significant 4 representatives, namely PMF, MF, the no-filter case, and the Miss-Matched Filter (MMF) with 1.5 times the sigma of MF. We observed the same trend in the simulation results as in 2D, i.e., PMF and MF offer the highest probability of detection. The MMF filter with a slightly different sigma than the simulated signal had performed slightly worse. Detection based on unfiltered values only has the lowest performance.

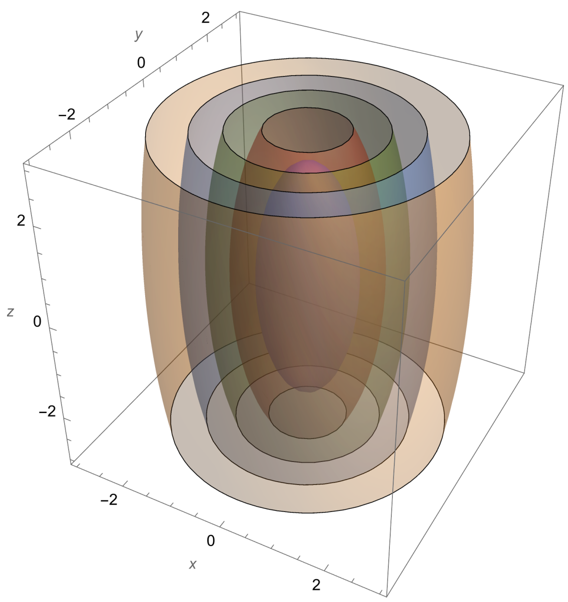

Supplementary Figure 4: A simple Gaussian model of a 3D PSF function asymmetric in the x-z axis resembling a rugby ball. Note that in order to plot the function of the three parameters $f(x,y,z)$ we need theoretically a 4D graph. Therefore, we use a contour plot, which represents the surfaces of the constancy of the function, for suitably chosen values of the constants. We plot the set of points (x,y,z) such that $f(x,y,z) = c$.

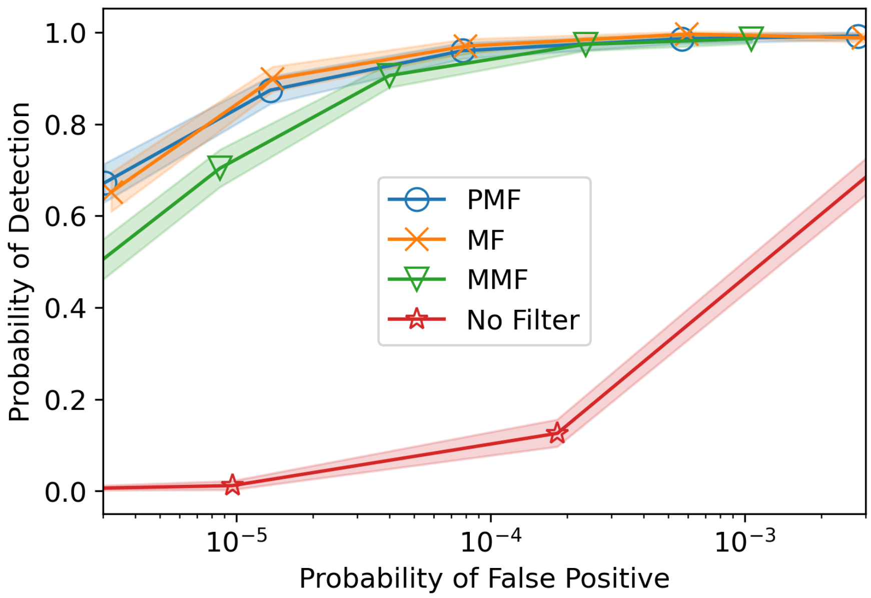

Supplementary Figure 5: The ROC performance curves for the detection of a randomly distributed molecule in a 3D datacube for the multiplane method with PMF, MF, Miss-Matched Filter (MMF), and no filter detection filters show a similar trend to that seen in the 2D ROC curves.

Supplementary Note 9: Experimental Dataset

9.1 Materials

60 nm gold nanoparticles (AuNP) were purchased from nanoComposix, USA, the product NanoXact Gold Spheres. DNA oligonucleotides and biotin-modified DNA oligonucleotides were purchased from Metabion. The list of all oligonucleotides and their sequence is in a separate supplementary Excel table (Supplementary Data 1). The M13mp18 scaffold was purchased from Tilibit. Chemicals purchased from Sigma-Aldrich: Bis(p-sulfonatophenyl)phenylphosphine dihydrate dipotassium salt (BSPP, no. 698539), Tris(2-carboxyethyl)phosphine hydrochloride (TCEP, no. 68957), Sodium dodecyl sulfate (SDS, no. L3771), Tris Acetate-EDTA buffer (TAE buffer, no. T8280), Albumin, biotin labeled bovine (BSA-biotin, no. A8549). Chemicals purchased from Thermo Fisher Scientific: NeutrAvidin Protein (no. 31000), Magnesium chloride (1 M, no. AM9530G), sodium chloride (5 M, no. AM9759). Methanol (M 03502) was purchased from p-Lab.

9.2 AuNP Functionalization

The procedure is based on an established protocol of Gür et al. with modifications [26]. To increase the nanoparticle stability at high concentrations, citrate-capped 60 nm AuNPs (0.05 mg/mL) are mixed with Bis(p-sulfonatophenyl)phenylphosphine (BSPP) to reach a concentration of 2.5 mM BSPP. The mixture is shaken overnight at room temperature. The solution is then centrifuged for 30 minutes at 10,000 x g. The supernatant is discarded, and the nanoparticles are redissolved in 1 mL freshly prepared 2.5 mM BSPP solution and 1 mL methanol. The solution is then again centrifuged at 10,000 x g for 30 min. The supernatant is discarded and 1 mL of 2.5 mM BSPP solution is added. The absorbance of the solution at 450 nm and the corresponding plasmon peak is measured on an Implen NanoPhotometer and the concentration is determined by calculating the mean value of the concentrations at the two wavelengths using differently sourced extinction coefficients [27], [28]. Thiolated ssDNA strands (linkers) with a T_{21} sequence are incubated with TCEP for one hour in a 1:250 ratio to reduce disulfide bonds. The solution is then added without further purification to the concentrated NP solution with a ratio of around 1:3500. This high excess ensures sufficient linker coverage on the AuNPs. To prevent aggregation of particles, sodium dodecyl sulfate (SDS) is added to a concentration of 0.2 %. To promote binding of oligonucleotides to the particle surface, salt-aging is performed. The mixture is brought to 0.75 M NaCl by adding 5 M NaCl solution in ten steps. After each addition, the solution is extensively vortexed, sonicated for 10 s, and left to incubate for 20 min. Afterwards, the solution is left on the shaker overnight at room temperature. The solution is then purified to remove excess unbound linkers by ultrafiltration in 100 kDa MWCO Amicon® Ultra centrifugal filters. Five rounds of centrifugation at 10,000 x g for 5 min are performed and after each round 400 μL of 1xTAE is added to the filter. After the last round, the retentate is recovered from the filter and the concentration is determined as described before. The functionalized nanoparticles are used immediately after purification.

9.3 DNA Origami Synthesis

Origami nanostructure sequences are designed and modified in caDNAno [29] and theoretically evaluated in terms of integrity and flexibility by oxDNA [30]. The 12-helix bundle structures are synthesized by mixing M13mp18 scaffold with the respective staple strands in a 1:10 ratio. The list of staple sequences and caDNAno sketch can be found in the Supplementary Excel table (Supplementary Data 1) and a json file (Supplementary Data 2). Modified staple strands for binding nanoparticles are added separately in a 1:15 ratio to ensure their incorporation. For microscopy immobilization, six biotin-functionalized oligonucleotides are incorporated at the bottom side of the structure. The mixture is brought to 1xTAE, 15 mM $MgCl_2$ and incubated in Biometra TAdvanced thermocycler (Jena Analytik) for around 25 h according to established protocols [31]. After completion, the structures are purified by five rounds of filtration in 100 kDa MWCO Amicon® Ultra centrifugal filters at 5000 x g for 5 min. After recovery of the retentate, the concentration is determined spectrophotometrically.

9.4 Origami-Particle Hybridization

AuNPs are mixed in a molar ratio of 1:N, with N being the number of nanoparticle binding sites per origami. The mixture is brought to 1xTAE, 10 mM $MgCl_2$ and is annealed from 40 °C to 25 °C over

90 min. The sample is then purified via gel electrophoresis in a 0.7 % agarose gel containing 1xTAE, 10 mM $MgCl_2$. The samples are mixed with 1xTAE, 10 mM $MgCl_2$, 30 % glycerol loading buffer in a 1:5 volume ratio and loaded into the gel. The gel is run for 60 min at 80 V and the bands of interest are cut and the sample is recovered via squeezing in parafilm-covered glass slides. After extraction, the samples a deposited on freshly cleaved mica and evaluated via AFM in a Bruker Dimension Icon in ScanAsyst mode.

9.5 SMLM Sample Preparation

Samples are prepared in μ-Slide 8 Well chambers (ibidi, Germany). To prepare the chamber for structure immobilization, it is first washed three times with 500 μL 1x TAE buffer. Then, 200 μL of biotinylated Bovine Serum Albumine (BSA-biotin, 1 mg/mL) is added to the chamber and incubated for 5 min on a shaker. Afterwards, the BSA-biotin solution is pipetted out of the well and it is again washed three times with 1xTAE. In the next step 200 μL of NeutrAvidin (1 mg/mL) is added to the well and incubated for around 30 min on the shaker. The NeutrAvidin solution is then again pipetted out and the well is flushed three times with immobilization buffer (IB, 1xTAE, 15 mM $MgCl_2$). Depending on the density of assemblies evaluated in AFM, the sample is diluted with immobilization buffer to yield a volume of 200 μL. This solution is then again added to the well and incubated for 5 min. The well is subsequently flushed carefully three times with IB.

9.6 SMLM Measurement

Microscopy measurements were performed using a home-built inverted microscope based on an Olympus IX71 body. A continuous wave laser (561 nm, Coherent, Sapphire) was used for sample excitation in TIRF configuration with an average laser power of 50 mW (measured in the sample plane). Light from the sample was collected by an oil immersion 100x objective (Olympus UAPON100XOTIRF, NA 1.49), and detected by an EMCCD camera (Andor iXon3 Ultra 897). A 655 nm long-pass filter (ET655lp, Chroma) was used in the detection path to remove the excitation beam. A baseline parameter of 100 Arbitrary Digital Units (ADU), quantum efficiency 90 %, and EM gain 300 were used to convert from ADU units to photon counts. To simulate low contrast measurement conditions, artificial background was added through the use of an additional spectrally broad microscope light source at two different intensities. For each dataset, 60 different 512x512 pixel Region Of Interests (ROIs) were measured, and then further cropped into non-overlapping 64x64 pixel patches. In this way, we created sequences of 3840 frames, containing about 5000 localizations.

Supplementary Note 10: Computational Complexity

Analysis of the computational complexity of the proposed algorithms can be divided into the filtering part and the background estimation part for probabilistic threshold.

10.1 Filtering Complexity

The proposed PMF filter is a linear filter whose asymptotic computational complexity is similar to that of other frequently occurring methods. Supplementary Table 1 summarizes the computational complexity of the considered methods, where we indicate whether a given filter can be implemented efficiently as a separable filter [11], or as a cascade of separable filters. For non-separable filters, we have the option of a direct implementation, a Fast Fourier Transform (FFT) implementation [11], and an incremental implementation [32]. The method of searching for the net gradient consists of calculating two separable gradient filters (Sobel filters of the size $K_1 \times K_1$), calculating the vector size (constant complexity), and calculating a separable arithmetic mean of kernel size $K_2 \times K_2$. Based on supplementary Table 1, we can conclude that all considered filtering methods have no worse than linear complexity and can be considered as fast. For the computational complexity of the algorithms in practice, it is especially important whether and in what form (e.g., support for parallel processing) implementations are available in the chosen software package.

**Filters**	**Linearity**	**Separability**	**Complexity per Pixel**
PMF, LoG	linear	non-separable	$O\left(K^2\right)$ [direct],<br>$O\left(\log_2 M\right)$ [FFT],<br>$O\left(1\right)$ [incremental]
MF	linear	separable	$O\left(K\right)$
DoG, DoA, BS, LG	linear	$2\times$ separable	$O\left(K\right)$
Net gradient search	non-linear	$2\times$ separable with K_1, $1\times$ separable with K_2	$O\left(\max(K_1, K_2)\right)$

Supplementary Table 1: Computational complexity of the considered methods. We express the complexity using big O notation of the number of Multiplies-and-ADDs (MADDs) [32], where we assume that the image size is $M \times M$ and the filter size is $K \times K$.

Supplementary Table 2 demonstrates specific computational times by supplementary speed tests in filtering of 128x128 image on a regular desktop PC (64bit AMD processor Ryzen 5, 5600x, 6-core@3.7GHz, 32GB RAM, no GPU acceleration). Note that unlike theoretical complexity, the speed test also tests the computational efficiency of the chosen programming language, the efficiency of its compiler, the availability of efficient algorithms in available libraries, the ability to employ a multi-core processing, and the speed of the operating system. The test has been performed using standard Python library functions `scipy.ndimage.convolve` for direct implementation and `scipy.signal.convolve` for FFT implementation not employing possible speed ups by separable filtering.

10.2 Complexity of Background Estimation

We express the computational complexity of the background estimation, in terms of asymptotic complexity in supplementary Table 3. Estimating the mean value (31 in the manuscript) from the neighborhood values in the form of a rectangular doughnut (depicted in Figure 3d) in the manuscript) can be implemented as a linear filtering of two separable box filters. Unlike the filtering with a disk-shaped kernel, which cannot be implemented as a separable filter. For the robust estimation using the median filter, there exist algorithms with linear or even constant complexity [33], [34]. However, these are usually not part of common computational libraries, and if they are, they do not always come with the possibility of using an arbitrary shaped filter kernel. The local estimation of the variance (35 in the manuscript) has similar complexity as the mean filter. Similar to filtering in the previous section, background estimation algorithms have no worse than linear complexity, but in practice it is crucial if efficient implementations are available in the chosen software package.

Similarly to the previous section, supplementary Table 4 presents run times of local background parameter estimates specific to Python implementation on our desktop PC. We can see that the time it

**Filters**	**Filter Size** K	**Direct Method** [ms]	**FFT** [ms]
PMF	11	1.2	0.4
MF	11	1.2	0.4
LoG	11	1.1	0.5
DoG	11	1.2	0.5
DoA	11	0.4	0.4
LG	11	0.5	0.4
BS $q = 3, s = 2$	13	1.8	0.5
BS $q = 3, s = 3$	25	9.2	0.6
Net-Gradient	$K_1 = 3, K_2 = 5$	1.3	-

Supplementary Table 2: Speed test of considered filtering methods of 128x128 image on regular PC confirms that PMF and MF filters are of comparable computational complexity as other filtering methods.

**Filters**	**Linearity**	**Kernel**	**Separability**	**Complexity per Pixel**
mean	linear	rectangle	$2\times$ separable	$O(K)$
		disk	non-separable	$O\left(K^2\right)$ [direct],<br>$O(\log_2 M)$ [FFT],<br>$O(1)$ [incremental]
median	non-linear	rectangle, disk	non-separable	$O\left(K^2 \log_2 K\right)$ [direct],<br>$O(K)$ [33], $O(1)$ [34]

Supplementary Table 3: Computational complexity of the methods locally estimating background.

takes to compute the local median depends significantly on the library function used. There is also a significant difference between the implementation of the local mean and median. Apparently, the library does not use the efficient implamentations described in references [33], [34] to compute the median.

**Local Background Estimates**	**Kernel Size** K	**Run Time** [ms]
mean (`scipy.ndimage.convolve`)	13	1.5
mean (`scipy.signal.fftconvolve`)	13	0.5
median (`scipy.ndimage.median_filter`)	13	21.3
median (`scipy.ndimage.generic_filter`)	13	249.0

Supplementary Table 4: Speed test of various methods for background parameter estimation available in standard Python library `scipy`.

Supplementary Note 11: Comparison to Deep Learning Methods

In this section, we place our method within the broader context of SMLM image processing by deep neural network models generally denoted as Deep Learning (DL) [35]. DL is being increasingly integrated into scientific discovery to augment and accelerate research, and tackle problems that might not have been possible using traditional analytical methods [36]. In the field of super-resolution microscopy DL is applied to a variety of tasks [37], becoming part of the toolbox of computational tools available for processing fluorescence microscope data [38].

Deep learning methods nowadays dominate various performance challenges over traditional analytical methods when evaluated over datasets carefully designed by experts in the field. In the context of SMLM, for example, DECODE method [39], currently leads the 2016 SMLM software challenge [2], especially in the scenario of densely emitting molecules and 3D data. These models offer advantages in terms of accuracy, resolution, and computational complexity.

An appealing feature of deep learning is their ability to learn task-specific representations from the data. Deep neural networks are so-called universal function approximators [37], capable of modeling very complex functions given sufficient and representative data. In the context of SMLM processing, DL can potentially replace any block in the SMLM processing pipeline illustrated in supplementary Figure 1 . Examples include a deep image segmentation algorithm that effectively replaces filtering and threasholding, [40], DECODE DL algorithm [39] outputs a list of localizations replacing block detection and localization, DL algorithms [41], [42], and [43] return super-resolution images effectively replacing the whole chain of operations plus super-resolution rendering algorithm. Similarly [44] proposes a sub-block of filtering that efficiently estimates and removes complex structured background from the data. More examples with the application of DL in SMLM are summarized also here [37].

Despite their abilities, DL models face several well-known challenges that limit their widespread adoption in mainstream practice, giving importance to classical analytical methods and their development (such as the one presented in this paper). One of the most significant issues is the lack of interpretability, often described as the "black box" problem [36] [45]. Unlike traditional analytical methods, where the rationale behind a result is clear, DL models typically provide little insight into how they arrive at their conclusion [46], being an area of active research [47]. The theory of deep learning is summarized e.g. [48], [49]. This lack of transparency is particularly concerning in high-stakes applications, where understanding the decision-making process is crucial.

Moreover, DL models are highly sensitive to the data used for training. They can perform unpredictably when confronted with new situations [46] or datasets that differ from those they were trained on [37], a phenomenon known as distributional shift. This sensitivity makes it challenging to ensure robust performance across diverse experimental conditions, and it raises concerns about generalizability [36]. For instance, DL methods may not perform consistently when applied to data from different microscopes or under varying imaging conditions [38]. Additionally, DL models can be susceptible to adversarial attacks, where subtle, often imperceptible changes to the input data can lead to significant errors in the output [38].

Most practical DL applications are in the cases where the penalty of incorrect prediction is small or can be independently verified, such as in new material discovery [50] or new matrix multiplication algorithm [51]. In SMLM for example the design of the optimal shape of the PSF function for densely emitting molecules in 3D [43], where its ideal properties can be verified retrospectively. Similarly, we can envision the design of an optimal detection filter for the case of dense emitters (in this paper we assume only sparse emitters) using DL.

In contrast to DL, our proposed optimal detection filter and probabilistic thresholding method are based on classical, analytical designs grounded in statistical hypothesis testing. The primary advantage of this approach is its predictability and interpretability. When the derivation conditions are met - such as the presence of dominant Poisson photon noise - the Neyman-Pearson theorem guarantees that our Poisson Matched Filter (PMF) offers the highest probability of detection for a given significance level. Similarly, our probabilistic thresholding ensures a controlled false positive rate under expected noise distributions.

When comparing the numerical complexity in the detection part of the SMLM algorithm, the DL segmentation is more numerically challenging than the linear filtering and threasholding presented in our method. Typical U-net-based [52] DL segmentation algorithms contain an image encoder that consists of a parallel bank of convolutional filters, i.e., itself involving many linear filtering operations. The computational complexity of some state-of-the-art DL segmentation models is far more complex than the

scale of linear filtering. For example, the number of parameters in the segment anything model [53] alone is over 600 million. DL models are typically less demanding and faster than the analytical solution if it is a full model that represents the entire chain of SMLM processing operations including the fitting of sub-pixel positions, the most computationally demanding part of the algorithm, see references [41], [42].

Supplementary Note 12: Extension to Multi-Molecule Detection Problem

Throughout the paper, we assume that the proposed algorithm is applied to a sufficiently sparse non-overlapping fluorescence signal which is part of the so-called single-emitter fitting problem. For the case of multiple overlapping signals (also denoted as a crowded field problem), the detection and localization algorithms often cooperate to deinterleave the overlapping signal. This type of detection is called a Multi-emitter Fitting Analysis (MFA). We refer to the overview of MFA methods to [54] and the references therein.

Of these MFA methods, we mention two methods that directly use single-emitter detection. One of them is data pre-processing using Haar wavelet transform [55], which essentially transforms dense-emitter data into sparse-emitter data. The second method is based on the sequential single molecule detection, reconstruction and elimination of the detected molecule to consecutively deinterleave the overlapping emitters one by one. These methods originally developed in astronomy such as the famous Högbom's CLEAN algorithm [56] and similar [8] were adopted in SMLM [57], [58], [59], [60]. For the performance comparison of individual MFA methods in SMLM we refer to [1] and [2].

Supplementary Note 13: Filter Kernel Visualization

Supplementary Figure 6 depicts the filters LoG, DoG, DoA, and MF. In all cases, we assume $\sigma = 1$. The comparison of the considered BS filters with PMF and MF filters is shown in supplementary Figure 7 and supplementary Figure 8.

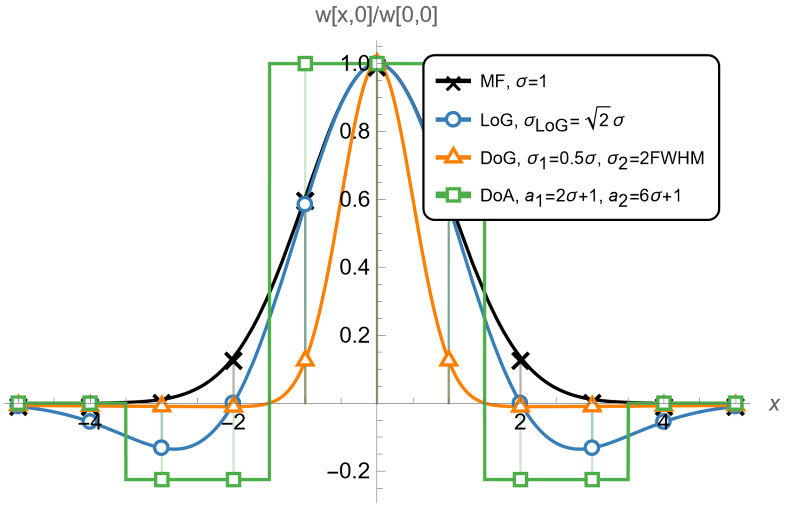

Supplementary Figure 6: Comparison of cross-sections of 2D MF, LoG, and DoG filters. The plots are normalized to unit-peak value. The markers describe sampled filter values w_{m0}. We see that the mainlobe of the LoG filter approximates MF very well, but differ in negative sidelobes which makes LoG, DoG, and DoA to be zero-mean filters.

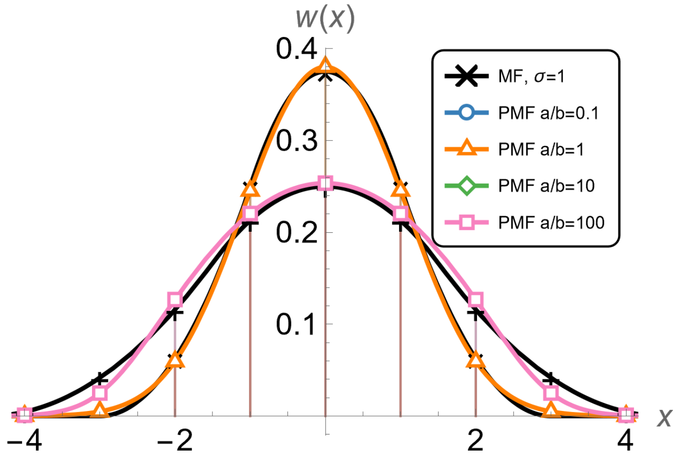

Supplementary Figure 7: Comparison of 1D normalized BS wavelet basis with 1D PMF filter. Markers correspond to the sampled filter values w_n. We see that PMF with a ratio of $a/b = 1$ is close to the 3rd order BS wavelet with scaling $s = 2$ and PMF $a/b = 100$ is close to BS with $q = 3, s = 3$.

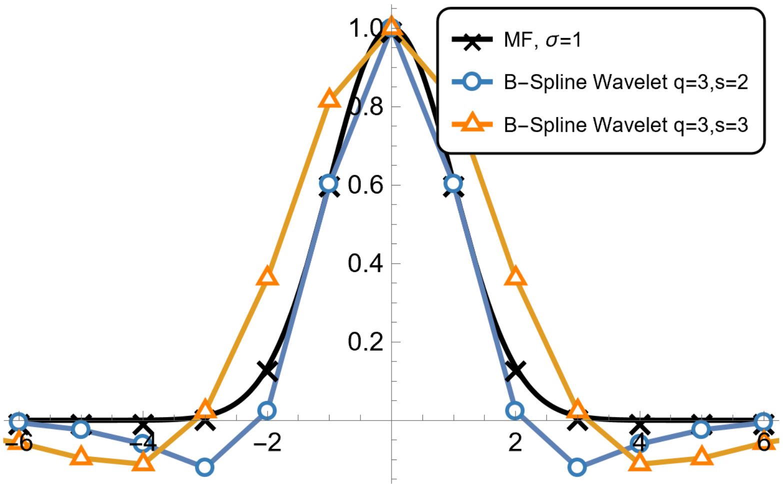

Supplementary Figure 8: Comparison of cross-sections of 2D MF filter with à trous BS wavelet filters. The plots are normalized to the unit-peak value.

Supplementary Note 14: Generalization for Molecule Detection in 3D

14.1 Detection in Multiplane

For the baseline Poisson noise model, the test hypotheses we choose from are

$$H_0 \text{ (signal absent)} : r_{lmn} \sim \mathscr{P}(b), \tag{22}$$

$$H_1 \text{ (signal present)} : r_{lmn} \sim \mathscr{P}(a s_{l-l_0,m-m_0,n-n_0} + b), \tag{23}$$

where r_{lmn} is the lmn-th voxel, a is the photon count, b is the mean background level and (x_0, y_0, z_0) is the molecule position. Symbol s_{lmn} denotes the 3D PSF and $\mathscr{P}$ is the Poisson distribution.

The derivation of the multiplane detector is identical as in the 2D case. Based on the noise model, we obtain the likelihood functions for the two hypotheses tested. The generalized likelihood-ratio test is a detector that will indicate a detection if the maximum of the test statistic over all possible positions of the molecule is greater than the detection threshold

$$\max_{l_0,m_0,n_0} \sum_{lmn} r_{lmn} w_{l_0-l,m_0-m,n_0-n} \overset{H_1}{>} \tau. \tag{24}$$

The summation operation is discrete 3D convolution which can be efficiently implemented by FFT using standard libraries such as `scipy.signal.convolve` in Python. For Poisson distributed noise, the optimal filter is the Poisson matched filter

$$w_{lmn} = \ln\left(1 + \frac{a}{b} s_{lmn}\right) \tag{25}$$

and for Gaussian distributed noise, the optimal filter is the matched filter $w_{lmn} = s_{lmn}$. Any analytical or measured model can be used as s_{lmn}.

14.2 Detection with z-parameterized PSF

In this case, one of the search parameters is the z-coordinate of the molecule, denoted here as l_0, which parameterizes the shape of the PSF.

The detection test resembles the aforementioned multiplane detector with the difference that it is a 2D convolution with a parametrized slice of the PSF function, namely

$$\max_{l_0,m_0,n_0} \sum_{mn} r_{mn} w_{m_0-m,n_0-n}(l_0) \overset{H_1}{>} \tau, \tag{26}$$

where $w_{mn}(l)$ is the l-th slice of the parametrized detection filter. For the Poisson noise model, the detection filter is

$$w_{mn}(l) = \ln\left(1 + \frac{a}{b} s_{mn}(l)\right) \tag{27}$$

and for Gaussian distributed noise, the optimal filter is the matched filter as proposed in [61]

$$w_{mn}(l) = s_{mn}(l). \tag{28}$$

Symbol $s_{mn}(l)$ denotes the l-th slice of parametrized PSF model.

Supplementary Figure 9: Performance of Detection Methods on 2D MT3 Dataset

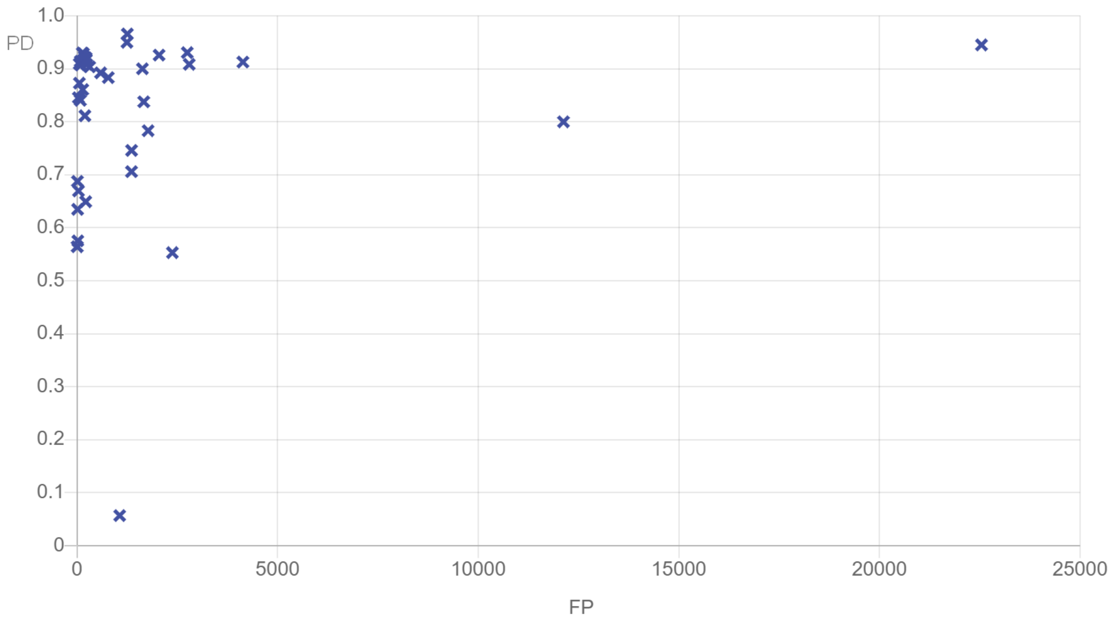

Supplementary Figure 9: This figure shows the dependence of the detection probability P_D on the number of false positive detections FP for the detection methods that participated in the SMLM localization software ground challenge 2016 [2]. Every cross is the performance of one method over dataset called MT3 containing 2D microtubule structures, which is publicly available at https://srm.epfl.ch/Challenge. We see that the amount of false positive detections considerably differs among the algorithms over the same dataset.

Supplementary Figure 10: Example Signal with a Uniform Background

The detection methods are tested in a constant background environment where a single randomly placed spot in Poisson noise is detected as illustrated in the following Figure.

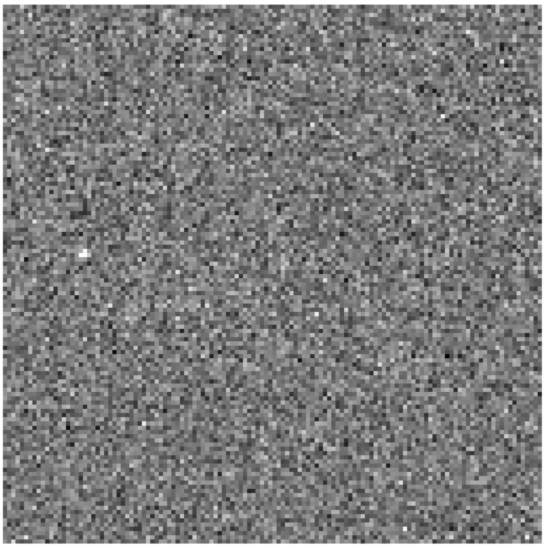

Supplementary Figure 10: Example of a randomly placed simulated fluorescent molecule on a uniform background with Poisson noise. The example considers a signal with a Gaussian type PSF with s.t.d. equal to 1 pixel with a photon count of 1000 ph and a uniform background with a mean value of 1000 photons. Frame size is 128×128 pixels.

Supplementary Figure 11: Jaccard Index Evaluation of Simulations with Uniform Background and Poisson Noise

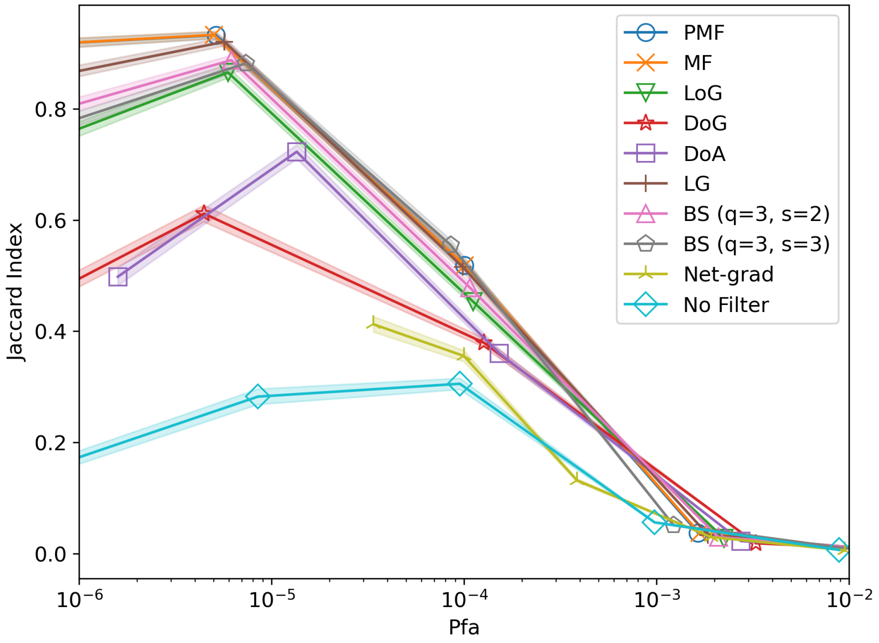

Supplementary Figure 11: Performance measured by Jaccard index highly depends on apriori distributions of H_0 and H_1 which is usually highly imbalanced (i.e. there are much more pixels with a background than a signal).

Supplementary Figure 12: Performance over Simulated Microtubule Dataset

- MT0.N2.LD dataset from SMLM software challenge 2016 [2]
- high noise, low density
- low background + EMCCD amplification noise gives non-Poisson, non-Gaussian noise, see Note 6 in SI

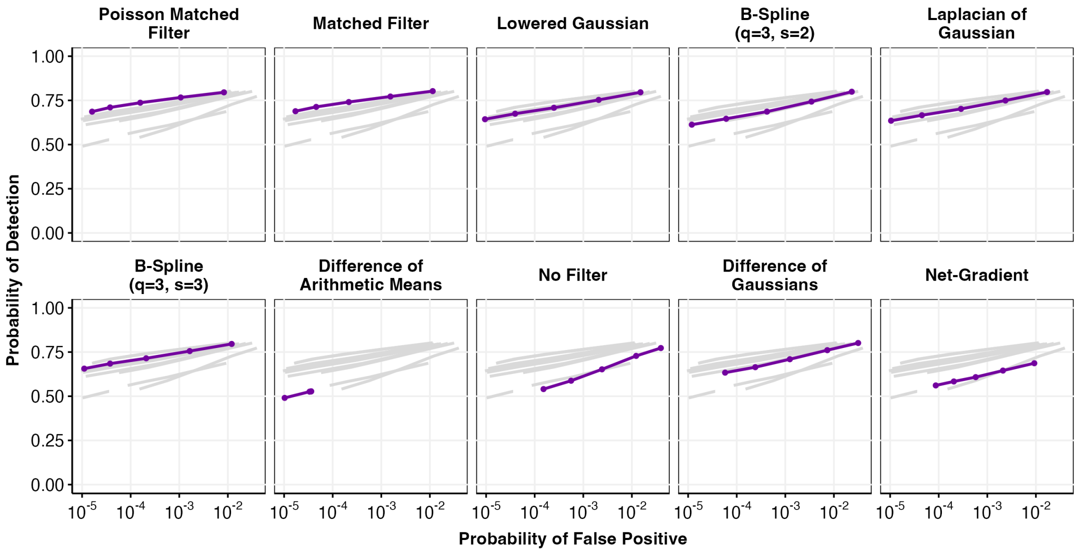

Supplementary Figure 12: Performance evaluation by ROC curves of the detection methods on Microtubule dataset from SMLM software challenge. PMF and MF are the best performers.

Supplementary Figure 13: Immobilization of DNA nanostructures for SMLM measurements

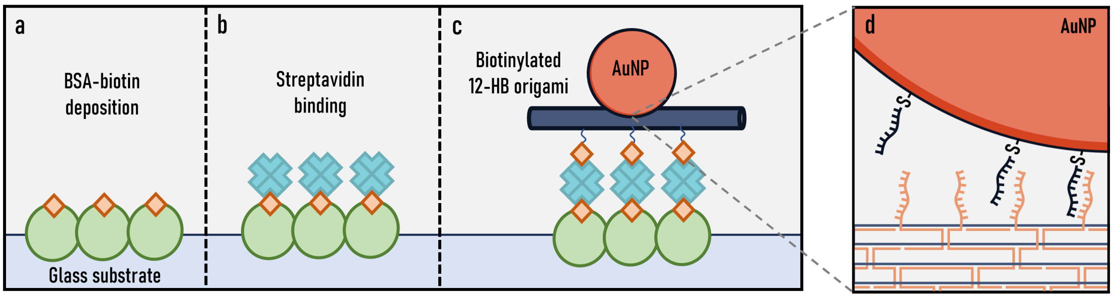

Supplementary Figure 13: Schematic visualization of DNA origami immobilization process with (a) biotinylated bovine serum albumin (BSA) adsorption and (b) subsequent streptavidin binding. DNA origami nanostructures in (c) contain biotin-functionalized staple strands which are utilized to immobilize the structures on the surface. AuNP are bound to DNA origami nanostructures via complimentary base pairing as shown in (d). AuNPs are functionalized with monothiolated DNA and DNA origami structures contain complimentary overhangs of single-stranded DNA.

Supplementary Figure 14: Gold Nanoparticles Attached to the DNA Origami

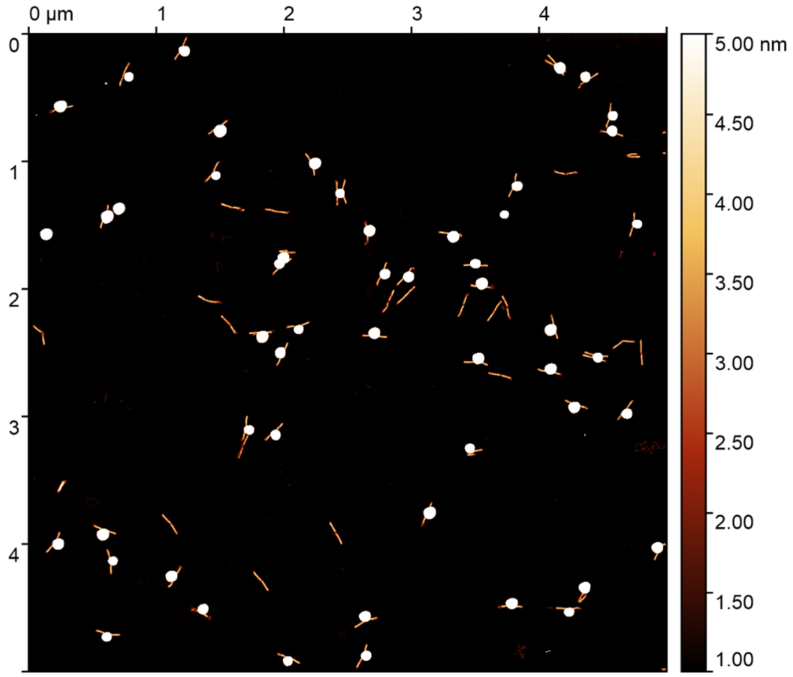

Supplementary Figure 14: AFM image of 60 nm gold nanoparticles attached to the DNA origami. The sample was used as a model sample to test the performance of the detection methods on experimental data.

Supplementary Figure 15: Impact of Integer Position Approximation

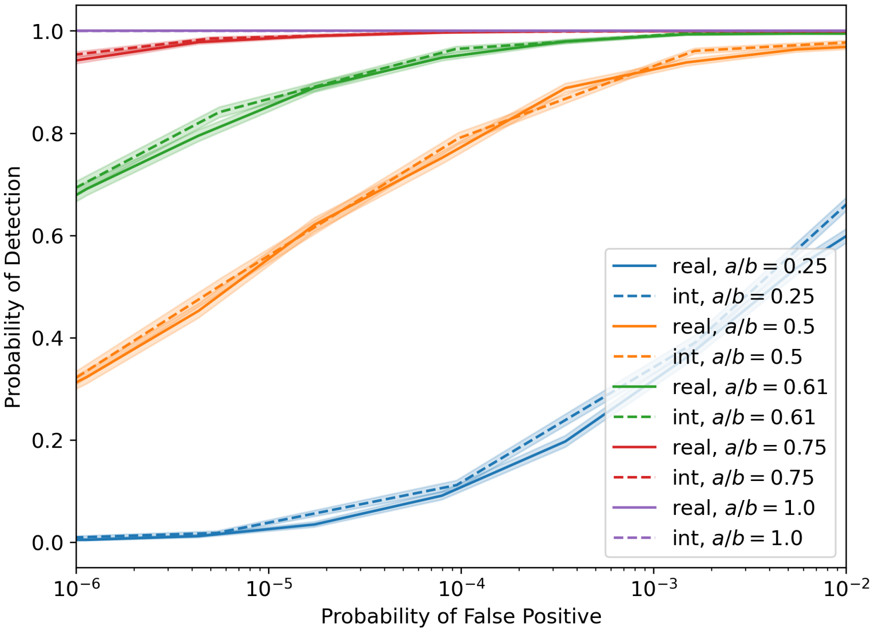

Supplementary Figure 15: Comparison of the performance of the PMF filter for the case when the coordinates of the fluorescent spots were generated as a random integer multiple of a pixel size and when they were generated as a random real number. The figure presents performance for different values of the signal-to-background ratio.

Supplementary Figure 16: Example Signal with a Varying Background

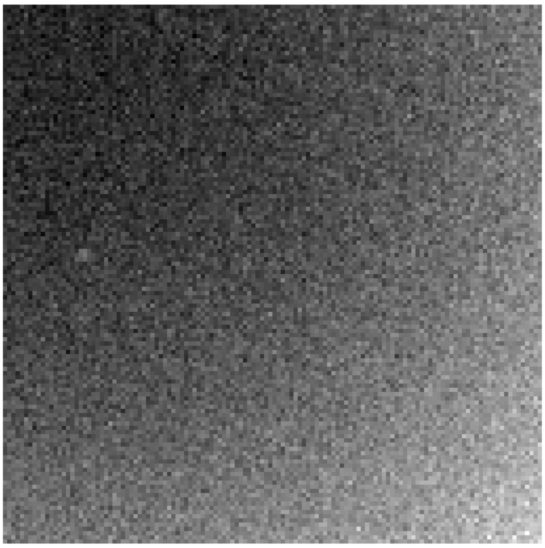

Supplementary Figure 16: Example of a randomly placed simulated fluorescent molecule on a non-uniform background with Poisson noise. The example considers a signal with a Gaussian type PSF with s.t.d. equal to 1 pixel with a photon count of 1000 ph and a parabolic non-uniform background with a mean value of 1000 photons and curvature of 10 pixels. Frame size is 128×128 pixels.

Supplementary Table 5: List of Synonyms

Terminology	Synonyms
Probability of detection	Probability of true positive, true positive rate, sensitivity, hit rate, recall, (statistical) power, 1- probability of type II error, 1- type II error rate
Probability of false positive	False positive rate, probability of false alarm, false alarm ratio, 1-specificity, significance level, fall-out, 1 - confidence level, test size, probability of type I error, type I error rate
Molecule detection	Spot detection [62], [5], particle detection [63], molecule identification, spot identification [58], ROI selection [64], ROI identification [65], finding candidate positions [66], finding approximate molecule position [67], emitter identification [3], segmentation [59], [18] (when local maximum search is not incorporated in the terminology)

Supplementary Table 5: List of terms used and their synonyms.

References

[1] D. Sage, H. Kirshner, T. Pengo, N. Stuurman, J. Min, S. Manley, and M. Unser, “Quantitative evaluation of software packages for single-molecule localization microscopy,” *Nature methods*, vol. 12, no. 8, pp. 717–724, 2015.

[2] D. Sage, T.-A. Pham, H. Babcock, T. Lukes, T. Pengo, J. Chao, R. Velmurugan, A. Herbert, A. Agrawal, S. Colabrese *et al.*, “Super-resolution fight club: assessment of 2D and 3D single-molecule localization microscopy software,” *Nature methods*, vol. 16, no. 5, pp. 387–395, 2019.

[3] A. Lee, K. Tsekouras, C. Calderon, C. Bustamante, and S. Pressé, “Unraveling the thousand word picture: an introduction to super-resolution data analysis,” *Chemical reviews*, vol. 117, no. 11, pp. 7276–7330, 2017.

[4] N. Chenouard, I. Smal, F. De Chaumont, M. Maška, I. F. Sbalzarini, Y. Gong, J. Cardinale, C. Carthel, S. Coraluppi, M. Winter *et al.*, “Objective comparison of particle tracking methods,” *Nature methods*, vol. 11, no. 3, pp. 281–289, 2014.

[5] I. Smal, M. Loog, W. Niessen, and E. Meijering, “Quantitative comparison of spot detection methods in fluorescence microscopy,” *IEEE transactions on medical imaging*, vol. 29, no. 2, 2010.

[6] P. Ruusuvuori, T. Äijö, S. Chowdhury, C. Garmendia-Torres, J. Selinummi, M. Birbaumer, A. M. Dudley, L. Pelkmans, and O. Yli-Harja, “Evaluation of methods for detection of fluorescence labeled subcellular objects in microscope images,” *BMC bioinformatics*, vol. 11, no. 1, pp. 1–17, 2010.

[7] E. Meijering, “Cell segmentation: 50 years down the road [life sciences],” *IEEE signal processing magazine*, vol. 29, no. 5, pp. 140–145, 2012.

[8] J.-L. Starck and F. Murtagh, *Astronomical image and data analysis*. Springer, 2007.

[9] D. Sage, F. R. Neumann, F. Hediger, S. M. Gasser, and M. Unser, “Automatic tracking of individual fluorescence particles: application to the study of chromosome dynamics,” *IEEE transactions on image processing*, vol. 14, no. 9, pp. 1372–1383, 2005.

[10] P. Milanfar, “A tour of modern image filtering: New insights and methods, both practical and theoretical,” *IEEE signal processing magazine*, vol. 30, no. 1, pp. 106–128, 2012.

[11] R. Gonzales and R. Woods, *Digital image processing*. Pearson, 2018.

[12] M. Sonka, V. Hlavac, and R. Boyle, *Image processing, analysis and machine vision*. Springer, 2013.

[13] S. Minaee, Y. Boykov, F. Porikli, A. Plaza, N. Kehtarnavaz, and D. Terzopoulos, “Image segmentation using deep learning: A survey,” *IEEE transactions on pattern analysis and machine intelligence*, vol. 44, no. 7, pp. 3523–3542, 2021.

[14] Z. Zou, K. Chen, Z. Shi, Y. Guo, and J. Ye, “Object detection in 20 years: A survey,” *Proceedings of the IEEE*, 2023.

[15] P. Virtanen, R. Gommers, T. E. Oliphant, M. Haberland, T. Reddy, D. Cournapeau, E. Burovski, P. Peterson, W. Weckesser, J. Bright *et al.*, “Scipy 1.0: fundamental algorithms for scientific computing in python,” *Nature methods*, vol. 17, no. 3, pp. 261–272, 2020.

[16] S. Kay, *Fundamentals of statistical signal processing: Detection theory*. Prentice Hall, 1998.

[17] R. J. Ober, E. S. Ward, and J. Chao, *Quantitative Bioimaging: An Introduction to Biology, Instrumentation, Experiments, and Data Analysis for Scientists and Engineers*. CRC Press, 2020.

[18] F. Huang, T. M. Hartwich, F. E. Rivera-Molina, Y. Lin, W. C. Duim, J. J. Long, P. D. Uchil, J. R. Myers, M. A. Baird, W. Mothes *et al.*, “Video-rate nanoscopy using sCMOS camera–specific single-molecule localization algorithms,” *Nature methods*, vol. 10, no. 7, pp. 653–658, 2013.

[19] C. S. Smith, N. Joseph, B. Rieger, and K. A. Lidke, “Fast, single-molecule localization that achieves theoretically minimum uncertainty,” *Nature methods*, vol. 7, no. 5, pp. 373–375, 2010.

[20] F. Aguet, D. V. De Ville, and M. Unser, "A maximum-likelihood formalism for sub-resolution axial localization of fluorescent nanoparticles," *Optics Express*, vol. 13, no. 26, pp. 10 503–10 522, 2005.

[21] M. Hirsch, R. J. Wareham, M. L. Martin-Fernandez, M. P. Hobson, and D. J. Rolfe, "A stochastic model for electron multiplication charge-coupled devices–from theory to practice," *PloS one*, vol. 8, no. 1, p. e53671, 2013.

[22] Z.-S. Ye and N. Chen, "Closed-form estimators for the gamma distribution derived from likelihood equations," *The American Statistician*, vol. 71, no. 2, pp. 177–181, 2017.

[23] M. A. Richards, *Fundamentals of radar signal processing*. McGraw-Hill Education, 2014.

[24] R. E. Blahut, *Theory of remote image formation*. Cambridge University Press, 2004.

[25] B. Hajj, M. El Beheiry, I. Izeddin, X. Darzacq, and M. Dahan, "Accessing the third dimension in localization-based super-resolution microscopy," *Physical Chemistry Chemical Physics*, vol. 16, no. 31, pp. 16 340–16 348, 2014.

[26] F. N. Gür, F. W. Schwarz, J. Ye, S. Diez, and T. L. Schmidt, "Toward self-assembled plasmonic devices: high-yield arrangement of gold nanoparticles on DNA origami templates," *ACS nano*, vol. 10, no. 5, pp. 5374–5382, 2016.

[27] W. Haiss, N. T. Thanh, J. Aveyard, and D. G. Fernig, "Determination of size and concentration of gold nanoparticles from UV-Vis spectra," *Analytical chemistry*, vol. 79, no. 11, pp. 4215–4221, 2007.

[28] A. Dolinnyi, "Extinction coefficients of gold nanoparticles and their dimers. dependence of optical factor on particle size," *Colloid Journal*, vol. 79, pp. 611–620, 2017.

[29] S. M. Douglas, A. H. Marblestone, S. Teerapittayanon, A. Vazquez, G. M. Church, and W. M. Shih, "Rapid prototyping of 3D DNA-origami shapes with caDNAno," *Nucleic Acids Research*, vol. 37, no. 15, pp. 5001–5006, Jun. 2009.

[30] E. Poppleton, R. Romero, A. Mallya, L. Rovigatti, and P. Šulc, "OxDNA.org: a public webserver for coarse-grained simulations of DNA and RNA nanostructures," *Nucleic Acids Research*, vol. 49, no. W1, pp. 491–498, 2021.

[31] M. Scheckenbach, T. Schubert, C. Forthmann, V. Glembockyte, and P. Tinnefeld, "Self-regeneration and self-healing in DNA origami nanostructures," *Angewandte Chemie International Edition*, vol. 60, no. 9, pp. 4931–4938, 2021.

[32] I. T. Young, J. J. Gerbrands, and L. J. Van Vliet, *Fundamentals of image processing*. Delft University of Technology Delft, 2007, vol. 841.

[33] T. Huang, G. Yang, and G. Tang, "A fast two-dimensional median filtering algorithm," *IEEE transactions on acoustics, speech, and signal processing*, vol. 27, no. 1, pp. 13–18, 1979.

[34] S. Perreault and P. Hébert, "Median filtering in constant time," *IEEE transactions on image processing*, vol. 16, no. 9, pp. 2389–2394, 2007.

[35] Y. LeCun, Y. Bengio, and G. Hinton, "Deep learning," *nature*, vol. 521, no. 7553, pp. 436–444, 2015.

[36] H. Wang, T. Fu, Y. Du, W. Gao, K. Huang, Z. Liu, P. Chandak, S. Liu, P. Van Katwyk, A. Deac *et al.*, "Scientific discovery in the age of artificial intelligence," *Nature*, vol. 620, no. 7972, pp. 47–60, 2023.

[37] L. Möckl, A. R. Roy, and W. Moerner, "Deep learning in single-molecule microscopy: fundamentals, caveats, and recent developments," *BIOMEDICAL OPTICS EXPRESS*, vol. 11, no. 3, pp. 1633–1661, 2020.

[38] C. Belthangady and L. A. Royer, "Applications, promises, and pitfalls of deep learning for fluorescence image reconstruction," *Nature methods*, vol. 16, no. 12, pp. 1215–1225, 2019.

[39] A. Speiser, L.-R. Müller, P. Hoess, U. Matti, C. J. Obara, W. R. Legant, A. Kreshuk, J. H. Macke, J. Ries, and S. C. Turaga, "Deep learning enables fast and dense single-molecule localization with high accuracy," *Nature methods*, vol. 18, no. 9, pp. 1082–1090, 2021.

[40] J. S. Danial, R. Shalaby, K. Cosentino, M. M. Mahmoud, F. Medhat, D. Klenerman, and A. J. Garcia Saez, "DeepSinse: deep learning-based detection of single molecules," *Bioinformatics*, vol. 37, no. 21, pp. 3998–4000, 2021.

[41] W. Ouyang, A. Aristov, M. Lelek, X. Hao, and C. Zimmer, "Deep learning massively accelerates super-resolution localization microscopy," *Nature biotechnology*, vol. 36, no. 5, pp. 460–468, 2018.

[42] E. Nehme, L. E. Weiss, T. Michaeli, and Y. Shechtman, "Deep-STORM: super-resolution single-molecule microscopy by deep learning," *Optica*, vol. 5, no. 4, pp. 458–464, 2018.

[43] E. Nehme, D. Freedman, R. Gordon, B. Ferdman, L. E. Weiss, O. Alalouf, T. Naor, R. Orange, T. Michaeli, and Y. Shechtman, "DeepSTORM3D: dense 3D localization microscopy and PSF design by deep learning," *Nature methods*, vol. 17, no. 7, pp. 734–740, 2020.

[44] L. Möckl, A. R. Roy, P. N. Petrov, and W. Moerner, "Accurate and rapid background estimation in single-molecule localization microscopy using the deep neural network BGnet," *Proceedings of the National Academy of Sciences*, vol. 117, no. 1, pp. 60–67, 2020.

[45] E. Korot, N. Pontikos, X. Liu, S. K. Wagner, L. Faes, J. Huemer, K. Balaskas, A. K. Denniston, A. Khawaja, and P. A. Keane, "Predicting sex from retinal fundus photographs using automated deep learning," *Scientific reports*, vol. 11, no. 1, p. 10286, 2021.

[46] Y. Bengio, G. Hinton, A. Yao, D. Song, P. Abbeel, T. Darrell, Y. N. Harari, Y.-Q. Zhang, L. Xue, S. Shalev-Shwartz *et al.*, "Managing extreme AI risks amid rapid progress," *Science*, vol. 384, no. 6698, pp. 842–845, 2024.

[47] S. Lapuschkin, S. Wäldchen, A. Binder, G. Montavon, W. Samek, and K.-R. Müller, "Unmasking Clever Hans predictors and assessing what machines really learn," *Nature communications*, vol. 10, no. 1, p. 1096, 2019.

[48] F. He and D. Tao, "Recent advances in deep learning theory," *arXiv preprint arXiv:2012.10931*, 2020.

[49] T. Poggio, A. Banburski, and Q. Liao, "Theoretical issues in deep networks," *Proceedings of the National Academy of Sciences*, vol. 117, no. 48, pp. 30 039–30 045, 2020.

[50] A. Merchant, S. Batzner, S. S. Schoenholz, M. Aykol, G. Cheon, and E. D. Cubuk, "Scaling deep learning for materials discovery," *Nature*, vol. 624, no. 7990, pp. 80–85, 2023.

[51] A. Fawzi, M. Balog, A. Huang, T. Hubert, B. Romera-Paredes, M. Barekatain, A. Novikov, F. J. R Ruiz, J. Schrittwieser, G. Swirszcz *et al.*, "Discovering faster matrix multiplication algorithms with reinforcement learning," *Nature*, vol. 610, no. 7930, pp. 47–53, 2022.

[52] T. Falk, D. Mai, R. Bensch, Ö. Çiçek, A. Abdulkadir, Y. Marrakchi, A. Böhm, J. Deubner, Z. Jäckel, K. Seiwald *et al.*, "U-Net: deep learning for cell counting, detection, and morphometry," *Nature methods*, vol. 16, no. 1, pp. 67–70, 2019.

[53] A. Kirillov, E. Mintun, N. Ravi, H. Mao, C. Rolland, L. Gustafson, T. Xiao, S. Whitehead, A. C. Berg, W.-Y. Lo *et al.*, "Segment anything," in *Proceedings of the IEEE/CVF International Conference on Computer Vision*, 2023, pp. 4015–4026.

[54] M. Fazel and M. J. Wester, "Analysis of super-resolution single molecule localization microscopy data: A tutorial," *AIP advances*, vol. 12, no. 1, 2022.

[55] R. J. Marsh, K. Pfisterer, P. Bennett, L. M. Hirvonen, M. Gautel, G. E. Jones, and S. Cox, "Artifact-free high-density localization microscopy analysis," *Nature methods*, vol. 15, no. 9, pp. 689–692, 2018.

[56] J. Högbom, “Aperture synthesis with a non-regular distribution of interferometer baselines,” *Astronomy and Astrophysics Supplement, Vol. 15, p. 417*, vol. 15, p. 417, 1974.

[57] S. J. Holden, S. Uphoff, and A. N. Kapanidis, “DAOSTORM: an algorithm for high-density super-resolution microscopy,” *Nature methods*, vol. 8, no. 4, pp. 279–280, 2011.

[58] R. Henriques, M. Lelek, E. F. Fornasiero, F. Valtorta, C. Zimmer, and M. M. Mhlanga, “QuickPALM: 3D real-time photoactivation nanoscopy image processing in ImageJ,” *Nature methods*, vol. 7, no. 5, pp. 339–340, 2010.

[59] F. Huang, S. L. Schwartz, J. M. Byars, and K. A. Lidke, “Simultaneous multiple-emitter fitting for single molecule super-resolution imaging,” *Biomedical optics express*, vol. 2, no. 5, 2011.

[60] A. Egner, C. Geisler, C. Von Middendorff, H. Bock, D. Wenzel, R. Medda, M. Andresen, A. C. Stiel, S. Jakobs, C. Eggeling *et al.*, “Fluorescence nanoscopy in whole cells by asynchronous localization of photoswitching emitters,” *Biophysical journal*, vol. 93, no. 9, pp. 3285–3290, 2007.

[61] A. Aristov, B. Lelandais, E. Rensen, and C. Zimmer, “ZOLA-3D allows flexible 3D localization microscopy over an adjustable axial range,” *Nature communications*, vol. 9, no. 1, pp. 1–8, 2018.

[62] U. Köthe, F. Herrmannsdörfer, I. Kats, and F. A. Hamprecht, “SimpleSTORM: a fast, self-calibrating reconstruction algorithm for localization microscopy,” *Histochemistry and Cell Biology*, vol. 141, pp. 613–627, 2014.

[63] J.-Y. Tinevez, N. Perry, J. Schindelin, G. M. Hoopes, G. D. Reynolds, E. Laplantine, S. Y. Bednarek, S. L. Shorte, and K. W. Eliceiri, “TrackMate: An open and extensible platform for single-particle tracking,” *Methods*, vol. 115, pp. 80–90, 2017.

[64] K. J. Martens, B. Turkowyd, and U. Endesfelder, “Raw data to results: a hands-on introduction and overview of computational analysis for single-molecule localization microscopy,” *Frontiers in bioinformatics*, vol. 1, p. 91, 2022.

[65] A. Small and S. Stahlheber, “Fluorophore localization algorithms for super-resolution microscopy,” *Nature methods*, vol. 11, no. 3, pp. 267–279, 2014.

[66] J. Ries, “SMAP: a modular super-resolution microscopy analysis platform for SMLM data,” *Nature Methods*, vol. 17, no. 9, pp. 870–872, 2020.

[67] M. Ovesný, P. Křížek, J. Borkovec, Z. Švindrych, and G. M. Hagen, “ThunderSTORM: a comprehensive ImageJ plug-in for PALM and STORM data analysis and super-resolution imaging,” *Bioinformatics*, vol. 30, no. 16, pp. 2389–2390, 2014.
