# Supplementary material for: Optimized molecule detection in localization microscopy with selected false positive probability
Source: Nat Commun. 2025 Jan 11;16:601. doi: 10.1038/s41467-025-55952-5 (PMC11724879; doi:10.1038/s41467-025-55952-5)
Supplement: Supplementary file 2 — Description of Additional Supplementary Files [file 41467_2025_55952_MOESM2_ESM.pdf]

Description of Additional Supplementary Files

Supplementary Data 1: List of staples for the DNA origami

Supplementary Data 2: caDNAno file containing the DNA origami design
